# Protective Effects of Pyrroloquinoline Quinone in Isoproterenol-Induced Myocardial Infarction in Rats

**DOI:** 10.3390/jcdd13100494

**Published:** 2026-10-01

**Authors:** Gülhan Ünlü, Kübra Tuğçe Kalkan, Seda Koçak

**Affiliations:** 1 Department of Pharmacology, Faculty of Medicine, Kirsehir Ahi Evran University, Kirsehir 40100, Türkiye; 2 Department of Histology and Embryology, Faculty of Medicine, Kirsehir Ahi Evran University, Kirsehir 40100, Türkiye; tugce.kalkan@ahievran.edu.tr; 3 Department of Physiology, Faculty of Medicine, Kirsehir Ahi Evran University, Kirsehir 40100, Türkiye

**Keywords:** myocardial infarction, isoproterenol, pyrroloquinoline quinone, rat

## Abstract

Cardiovascular diseases remain the leading cause of mortality worldwide, with myocardial infarction (MI) representing one of the most severe manifestations. Oxidative stress, inflammation, and apoptosis are major contributors to myocardial injury. Pyrroloquinoline quinone (PQQ), a potent redox cofactor, has demonstrated antioxidant and cytoprotective properties; however, its effects on isoproterenol (ISO)-induced myocardial injury remain unclear. This study investigated the cardioprotective effects of PQQ in an ISO-induced MI rat model using biochemical, histopathological, and immunohistochemical analyses. Thirty-two male Wistar rats were divided into four groups: Control, PQQ, ISO-MI, and ISO-MI+PQQ. MI was induced by subcutaneous ISO administration (85 mg/kg) for two consecutive days, while PQQ (3 mg/kg) was administered orally for 14 days. ISO significantly increased Troponin T levels and caused myocardial necrosis, edema, inflammation, and fibrosis. PQQ treatment significantly attenuated myocardial damage, reduced Troponin T levels, increased CAT activity, and improved oxidative balance. Immunohistochemical findings showed decreased BAX expression in the PQQ-treated group. These findings indicate that PQQ may have beneficial effects in this experimental model; however, further studies are required to clarify these effects and their potential clinical relevance.

## 1. Introduction

Cardiovascular diseases (CVDs) are a broad group of disorders affecting the heart and blood vessels and remain one of the most important causes of death and disability worldwide [1]. Among these disorders, ischemic heart disease (IHD) develops when coronary blood flow becomes insufficient to meet the metabolic demands of the myocardium. Reduced oxygen supply to cardiac tissue may eventually result in myocardial infarction (MI), angina, arrhythmias, or heart failure [2].

Oxidative stress is considered one of the principal mechanisms involved in the progression of cardiovascular disorders. Excessive production of reactive oxygen species (ROS) disrupts cellular homeostasis and contributes to lipid peroxidation, protein oxidation, mitochondrial dysfunction, and cell death. In recent years, increasing attention has been directed toward therapeutic agents capable of reducing oxidative damage and preserving myocardial integrity. In addition, disturbances in protein quality control and accumulation of damaged proteins in cardiomyocytes have been associated with the development of cardiac dysfunction and heart failure [3,4]. ROS are also known to influence intracellular signaling pathways that regulate cardiomyocyte survival, inflammation, and apoptosis [5].

Although substantial improvements have been achieved in the prevention and treatment of MI through pharmacological and interventional approaches, MI-related mortality and morbidity remain significant clinical concerns [6,7]. Following ischemic injury, the myocardium undergoes a series of pathological alterations characterized by inflammation, oxidative stress, hypoxia, and ventricular remodeling [8]. Persistent imbalance between oxidant production and antioxidant defense systems further aggravates tissue injury and contributes to progressive cardiac dysfunction [9].

Isoproterenol (ISO) is a synthetic catecholamine and non-selective β-adrenergic agonist that stimulates both β1 and β2 receptors [10,11]. While low doses of ISO may exert positive chronotropic and inotropic effects, administration of high doses causes excessive myocardial workload and oxygen consumption, resulting in myocardial necrosis resembling infarction [12]. Experimental studies have shown that ISO-induced cardiac injury shares several pathological and biochemical features with human MI, including oxidative stress, inflammation, and apoptosis [13]. For this reason, ISO-induced myocardial injury is widely used as an experimental model in cardiovascular research.

Pyrroloquinoline quinone (PQQ) is a biologically active redox compound initially identified as a cofactor in bacterial enzymes [14,15]. Subsequent studies demonstrated its presence in mammalian tissues and its involvement in cellular growth, mitochondrial function, and tissue regeneration [14,15,16]. PQQ has attracted considerable interest because of its strong antioxidant potential and its ability to protect cells against oxidative damage [17]. It has been reported to scavenge reactive oxygen species, support mitochondrial function, and regulate cellular redox balance [17,18].

Recent investigations have suggested that PQQ may also exert beneficial effects on the cardiovascular system. Experimental findings indicate that PQQ improves endothelial function, suppresses inflammatory responses, and reduces ischemia-related tissue damage [19,20]. Since cardiac tissue requires continuous and high levels of ATP production, impairment in mitochondrial function may severely affect myocardial performance. Studies have further demonstrated that PQQ supplementation enhances resistance to ischemic injury and provides protection against mitochondrial oxidative damage [21,22,23]. However, despite these promising findings, further characterization of the effects of PQQ in ISO-induced myocardial injury may provide additional insights, particularly through the integrated assessment of hemodynamic, biochemical, oxidative stress, histopathological, and apoptosis-related parameters within the experimental model.

Although the cardioprotective effects of PQQ have been investigated in various experimental models, its effects on ISO-induced myocardial injury, a catecholamine-mediated model characterized by oxidative stress, myocardial necrosis, and hemodynamic alterations, remain insufficiently characterized. In particular, the effects of PQQ on biochemical, oxidative stress, hemodynamic, histopathological, and apoptosis-related parameters have not been comprehensively evaluated within the same experimental model. Considering the important contribution of oxidative stress and inflammation to myocardial injury, the present study aimed to provide an integrated assessment of the potential cardioprotective effects of PQQ in ISO-induced myocardial injury in rats.

## 2. Materials and Methods

### 2.1. Study Design and Ethical Approval

In this study, the cardioprotective effects of PQQ were investigated in an ISO-induced MI model. The potential protective roles of PQQ against inflammation and myocardial injury were evaluated using biochemical, histopathological, and immunohistochemical methods.

The experiment was conducted at the Experimental Research Center of Kırşehir Ahi Evran University. Ethical approval for the study was obtained from the Kırşehir Ahi Evran University Local Ethics Committee for Animal Experiments (Approval No:13/2; Date: 25 June 2025) and performed in accordance with the ARRIVE guidelines and the National Institutes of Health Guide for the Care and Use of Laboratory Animals.

### 2.2. Experimental Animals and Housing Conditions

The study initially included 32 male Wistar albino rats, with eight animals allocated to each of the four experimental groups. During the experimental period, one animal in the ISO-MI group and one animal in the ISO-MI+PQQ group died. Therefore, the final sample sizes were *n* = 8 for the Control and PQQ groups and *n* = 7 for the ISO-MI and ISO-MI+PQQ groups. The rats were 8–10 weeks old and weighed between 300 and 350 g. They were kept in an autonomously climate-controlled room with a 12-h light/dark cycle and a temperature of 22–24 °C throughout the experiment. Standard pellet feed and unlimited access to tap water were provided to the animals. Each cage contained 3–5 rats.

### 2.3. Experimental Groups

The animals were randomly assigned into four groups using simple randomization, each consisting of 8 rats. The number of animals in each group was determined based on a previous study using a similar experimental model [24].

- Group 1 (Control): Rats received standard pellet chow and tap water for 14 days without any additional treatment.
- Group 2 (PQQ): Rats received 3 mg/kg [22] of PQQ (Cat No: GC44793, GlpBio Technology, Montclair, CA, USA) by oral gavage for 14 consecutive days [25] in addition to standard feed and water.
- Group 3 (ISO-MI): After 14 days of standard feeding, injection of ISO subcutaneously caused MI (Lot: 4122564, Merck, Germany) at a dose of 85 mg/kg for two consecutive days [26].
- Group 4 (ISO-MI+PQQ): Rats received 3 mg/kg of PQQ orally for 14 consecutive days. On days 15 and 16, MI was induced by subcutaneous administration of ISO (85 mg/kg).

### 2.4. Body Weight Measurements

Body weight was measured and recorded on experimental days 1, 5, 9, and 14. At the end of the treatment period, the rats were anesthetized with thiopental sodium, and the experiment was terminated.

### 2.5. Administration of Pyrroloquinoline Quinone

Previous studies had reported different doses of PQQ ranging from 3 to 15 mg/kg [19,22]. In the present study, a dose of 3 mg/kg, previously shown to exert protective effects, was administered by oral gavage for 14 consecutive days.

### 2.6. Induction of Myocardial Infarction

The rats were given 85 mg/kg ISO subcutaneously to cause MI. One rat from the ISO-MI group and one from the ISO-MI+PQQ group perished following the administration of ISO at a dose of 85 mg/kg. The animals received intraperitoneal anesthesia with 50 mg/kg thiopental sodium at the conclusion of the experiment. A thoracotomy was carried out while the animals were under thiopental anesthesia, and blood was drawn straight from their hearts. The animals were put to death by cervical dislocation after tissue and blood samples were obtained while they were under general anesthesia.

### 2.7. Biochemical Analysis

Blood samples were allowed to clot at room temperature for approximately 30–40 min in gel-containing vacuum tubes. The serum was separated from the samples by centrifuging them for 15 min at 3000 rpm. Before being subjected to biochemical assays, the serum samples were kept at −80 °C.

Serum troponin T (TNNT-2; BT Lab, Shanghai, China, Cat. No. E0306Ra) and B-type natriuretic peptide (BNP; Elabscience Biotechnology Co. Ltd., Wuhan, China, Cat. No. E-EL-R0126) levels were measured using competitive enzyme-linked immunosorbent assay (ELISA) kits according to the manufacturers’ instructions. Absorbance was measured at 450 nm using a BIO-TEK ELx800 microplate reader. Serum malondialdehyde (MDA; Otto Scientific, Ankara, Türkiye, Cat. No. Otto1001) levels were determined using a thiobarbituric acid (TBA)-based colorimetric method, with absorbance measured at 532 nm. Superoxide dismutase (SOD; Otto Scientific, Cat. No. Otto284627) and catalase (CAT; Otto Scientific, Cat. No. Otto587794) activities were measured in cardiac tissue homogenates using commercially available colorimetric assay kits according to the manufacturer’s instructions. SOD and CAT activities were determined at 505 nm and 405 nm, respectively, and expressed as U/mL.

### 2.8. Blood Pressure Measurement

Tail-cuff plethysmography (MAY NIBP250, Turkey) was used to measure blood pressure before and after the experimental period in order to confirm ISO-induced MI. After 20 min of restraint, cuffs were placed on each animal’s tail, and blood pressure readings were taken. Five consecutive readings were taken at intervals of one minute for each animal. Systolic blood pressure, diastolic blood pressure, heart rate, and mean arterial pressure were determined by averaging the remaining three observations after the highest and lowest values were eliminated [27].

MAP was calculated using the following formula:

MAP = DP + (SP − DP)/3

where SP represents systolic pressure, and DP represents diastolic pressure.

### 2.9. Cardiac Histopathology Evaluation

Histopathological evaluation was performed by a single experienced histologist who was blinded to the experimental group allocation, using predefined semi-quantitative scoring criteria [28].

To maintain tissue morphology, cardiac tissues from each experimental group were fixed in 10% neutral buffered formaldehyde for 72 h. Following fixation, the samples were cleaned in xylene, dehydrated using graded alcohol solutions, rinsed under running water, and embedded in paraffin blocks. A microtome was used to segment paraffin-embedded tissues at a thickness of 5 μm for histological analysis. After deparaffinizing the sections in xylene, they were rehydrated using a declining alcohol series (100%, 96%, 80%, 70%, and 50%) and then cleaned with distilled water. The general histopathological architecture of cardiac tissue was evaluated using hematoxylin and eosin (H&E) staining. Cardiomyocyte necrosis, interstitial edema, and inflammatory cell infiltration were used to assess myocardial damage in these sections. To identify fibrotic scar regions in the cardiac tissue, Masson’s Trichrome (MT) staining was also carried out. After completion of staining procedures, the sections were dehydrated through an ascending alcohol series, cleared with xylene, and mounted with Entellan under a coverslip. All histological preparations were examined using a light microscope (Nikon Eclipse Si, Tokyo, Japan). For each sample, ten randomly selected microscopic fields were evaluated. Histopathological alterations, including cardiomyocyte necrosis, interstitial edema, and inflammatory cell infiltration, were scored semi-quantitatively. Fibrotic scar areas observed in MT-stained sections were also evaluated. The severity of the histopathological findings was graded using a four-point scoring system: 0 (none), 1 (mild), 2 (moderate), and 3 (severe).

### 2.10. Immunohistochemical Evaluation

Immunohistochemical staining was performed to determine the expression levels of apoptosis-related markers BAX and BCL-2 in cardiac tissue sections. For this purpose, paraffin-embedded tissue samples obtained from all experimental groups were used. Sections with a thickness of 5 μm were mounted on poly-L-lysine–coated slides and prepared for immunohistochemical analysis. The sections were first rinsed in distilled water after being deparaffinized in xylene and rehydrated using a succession of graded alcohols. The sections were heated in citrate buffer using a microwave oven to achieve antigen retrieval. Phosphate-buffered saline (PBS) was used to wash the sections once they had cooled to room temperature. The slices were incubated with 3% hydrogen peroxide (H_2_O_2_) to inhibit endogenous peroxidase activity. The sections were treated with blocking serum for ten minutes at room temperature to stop nonspecific antibody binding. The sections were then treated with primary antibodies against BAX (BAX 50599-2-Ig proteintech 1/2000) and BCL-2 (BCL-2 26593-1-ap proteintech 1/400) at the proper dilutions for a whole night at +4 °C. The sections were treated with a biotinylated secondary antibody in accordance with the manufacturer’s instructions after being cleaned with PBS. The streptavidin-biotin peroxidase technique and an HRP detection kit (UltraVision Polyvalent (Rabbit-Mouse) Horseradish Peroxidase Kit, Thermo Fisher Scientific, Waltham, MA, USA) were used to visualize immunoreactivity. The chromogen utilized to create a brown reaction product at the antigen–antibody binding sites was diaminobenzidine (DAB). The sections were then cleaned in xylene, mounted with Entellan, dehydrated using graded alcohol solutions, and counterstained. A light microscope (Nikon Eclipse Si, Tokyo, Japan) was used to view each stained segment. Images were obtained from randomly selected microscopic fields for each sample. The intensity of BAX and BCL-2 immunoreactivity was analyzed using ImageJ software(Version 1.52a National Institutes of Health, Bethesda, MD, USA), and the percentage of DAB-positive staining areas was determined using the color thresholding function of the program.

### 2.11. Statistical Analyses

Statistical analyses were performed using GraphPad Prism version 10.0. Data distribution normality was evaluated using the Shapiro–Wilk test. Parametric data were analyzed using one-way analysis of variance (ANOVA) followed by a Bonferroni post hoc test, whereas non-parametric data were analyzed using the Kruskal–Wallis test followed by Dunn’s post hoc test.

Parametric data were expressed as mean ± standard error of the mean (SEM), whereas non-parametric data were presented as median (minimum–maximum). Statistical significance was set at *p* < 0.05. Significance levels are indicated as * *p* < 0.05, ** *p* < 0.01, *** *p* < 0.001.

## 3. Results

### 3.1. Evaluation of Weight Changes in Groups

**Figure 1:**
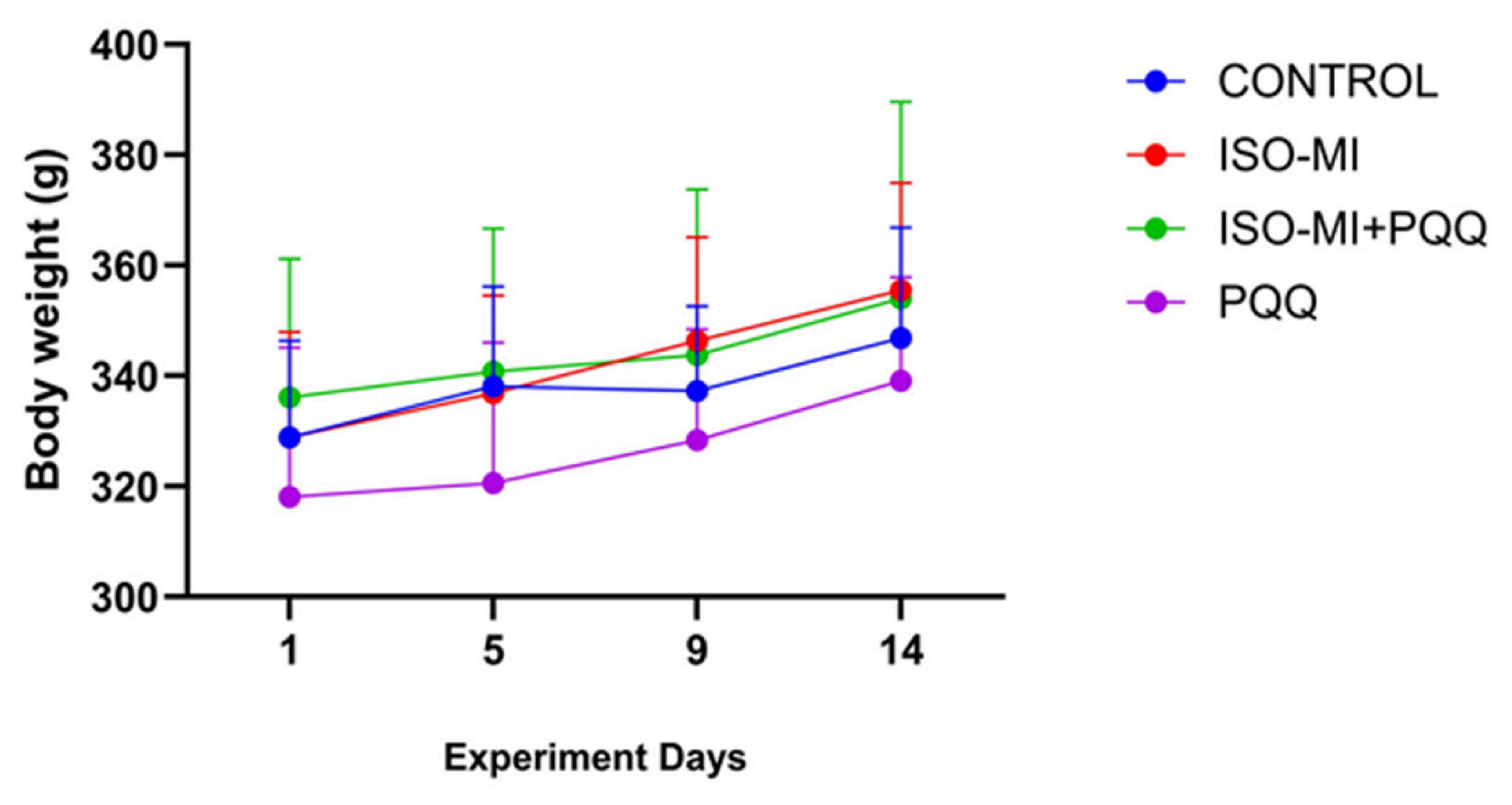
Changes in body weights of the groups across the experimental days.

The body weights of the experimental animals increased significantly over time; however, no statistically significant differences were observed between the groups. The body weights of the groups on days 1 to 14 are presented in Figure 1.

### 3.2. Assessment of Serum Biochemical Markers

**Figure 2:**
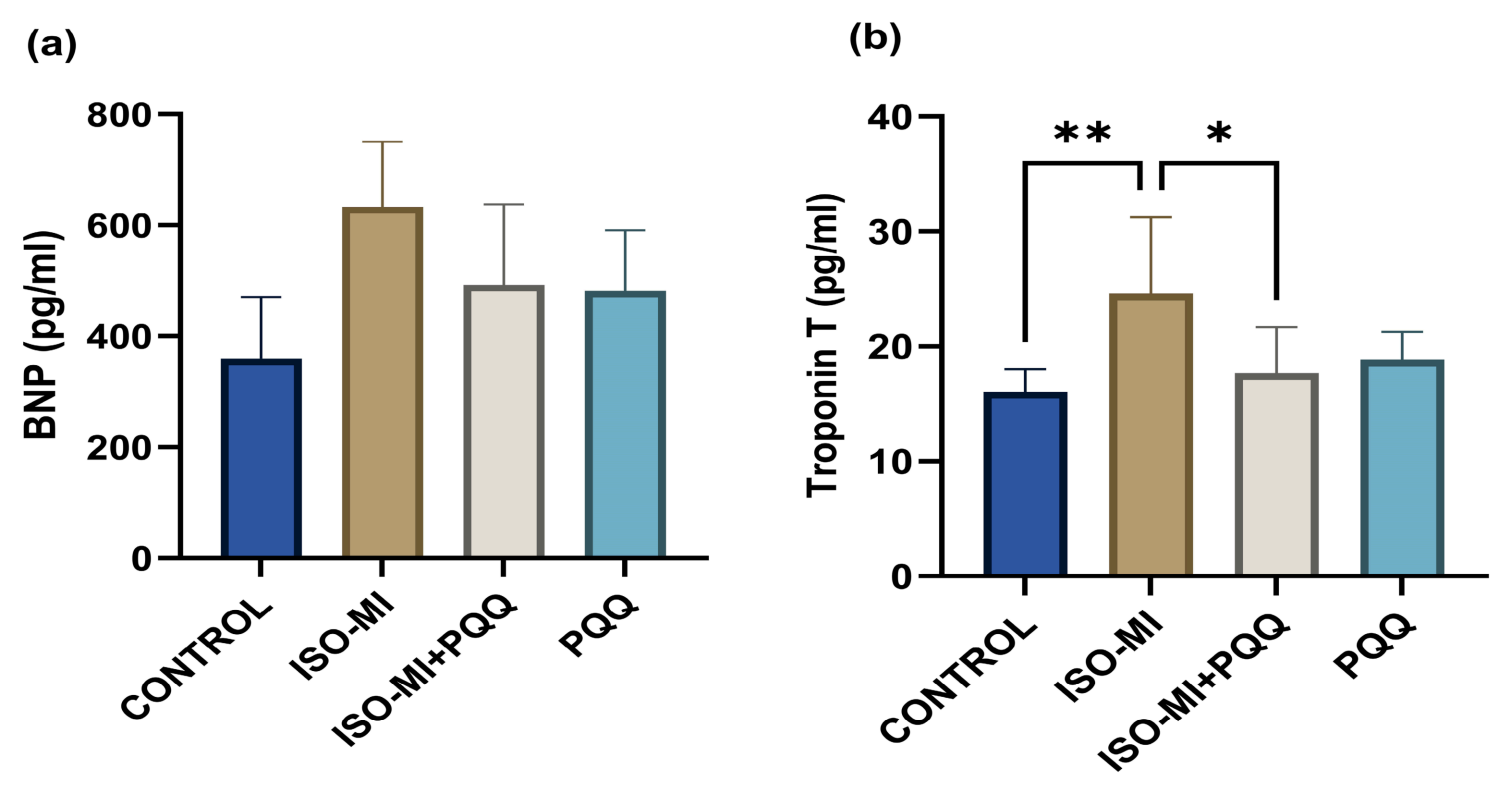
Effects of PQQ on (**a**) BNP and (**b**) Troponin T levels across experimental groups. * *p* < 0.05, ** *p* < 0.01.

**Figure 3:**
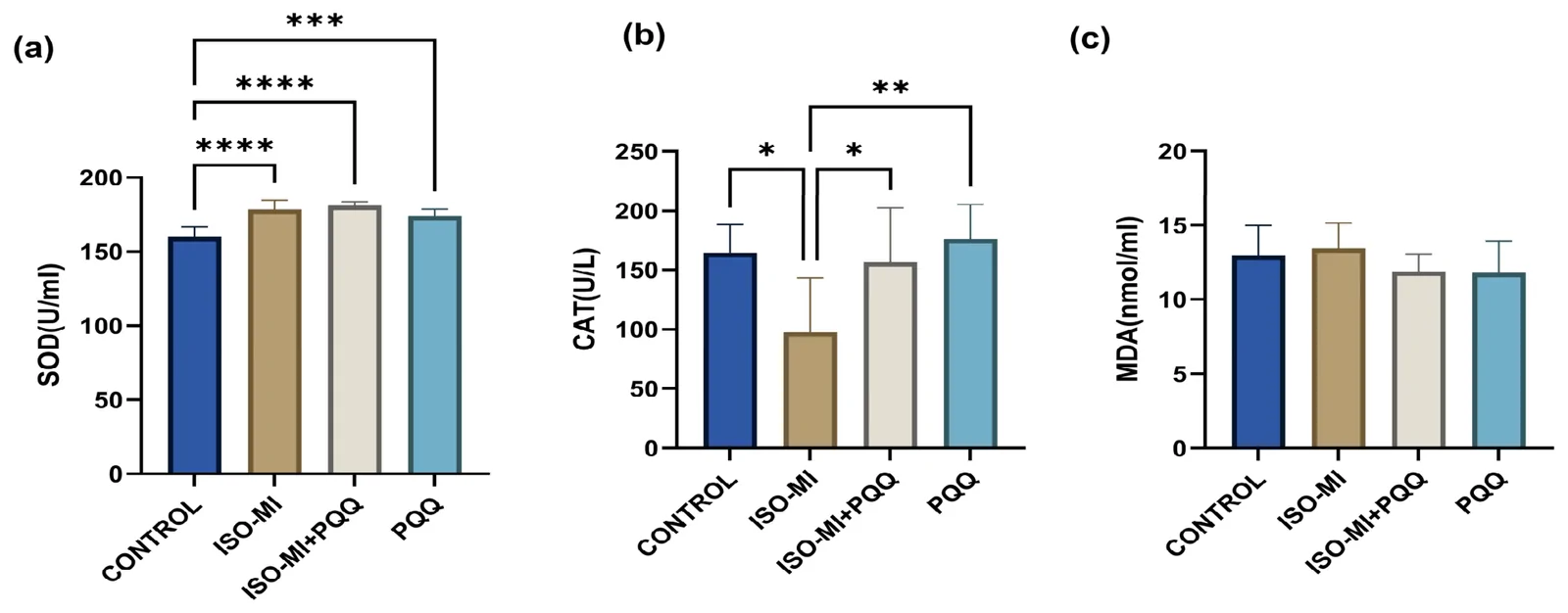
Effects of PQQ on (**a**) SOD and (**b**) CAT and (**c**) MDA levels across experimental groups. * *p* < 0.05, ** *p* < 0.01, *** *p* < 0.001, **** *p* < 0.0001.

In our study, the biochemical parameters of the groups were evaluated and the effects of ISO-induced MI and PQQ administration on Troponin T, BNP, MDA, SOD and CAT levels were examined. The biochemical findings are summarized in Table 1 and illustrated in Figure 2 and Figure 3.

### 3.3. Evaluation of Serum BNP and Troponin T Levels

There was no statistically significant difference in BNP levels among the experimental groups (Figure 2a).

Serum Troponin T levels were compared among the groups, revealing a significant increase in the ISO-MI group (24.6 ± 2.7 pg/mL) compared to the control group (16.1 ± 0.8 pg/mL) (*p* = 0.01). In the ISO-MI+PQQ group, Troponin T levels (17.7 ± 1.6 pg/mL) were significantly reduced compared to the ISO-MI group and approached control values (*p*= 0.04). These findings indicate that PQQ administration significantly attenuates the increase in Troponin T associated with ISO-induced MI (Figure 2b).

### 3.4. Evaluation of Serum SOD, CAT and MDA Levels

Serum SOD levels were significantly higher in the ISO-MI, ISO-MI+PQQ, and PQQ groups compared with the control group (*p* < 0.0001, *p* < 0.0001, and *p* = 0.0008, respectively). No significant difference in SOD levels was observed among the ISO-MI, ISO-MI+PQQ, and PQQ groups (Figure 3a).

Serum CAT levels were significantly lower in the ISO-MI group compared with the control group (*p* = 0.0118). CAT levels were significantly higher in the ISO-MI+PQQ group compared with the ISO-MI group (*p* = 0.04 (Figure 3b).

MDA levels (Figure 3c) were slightly elevated in the ISO-MI group compared to the control group; however, no statistically significant differences were observed among the groups.

### 3.5. Blood Pressure Measurements

**Figure 4:**
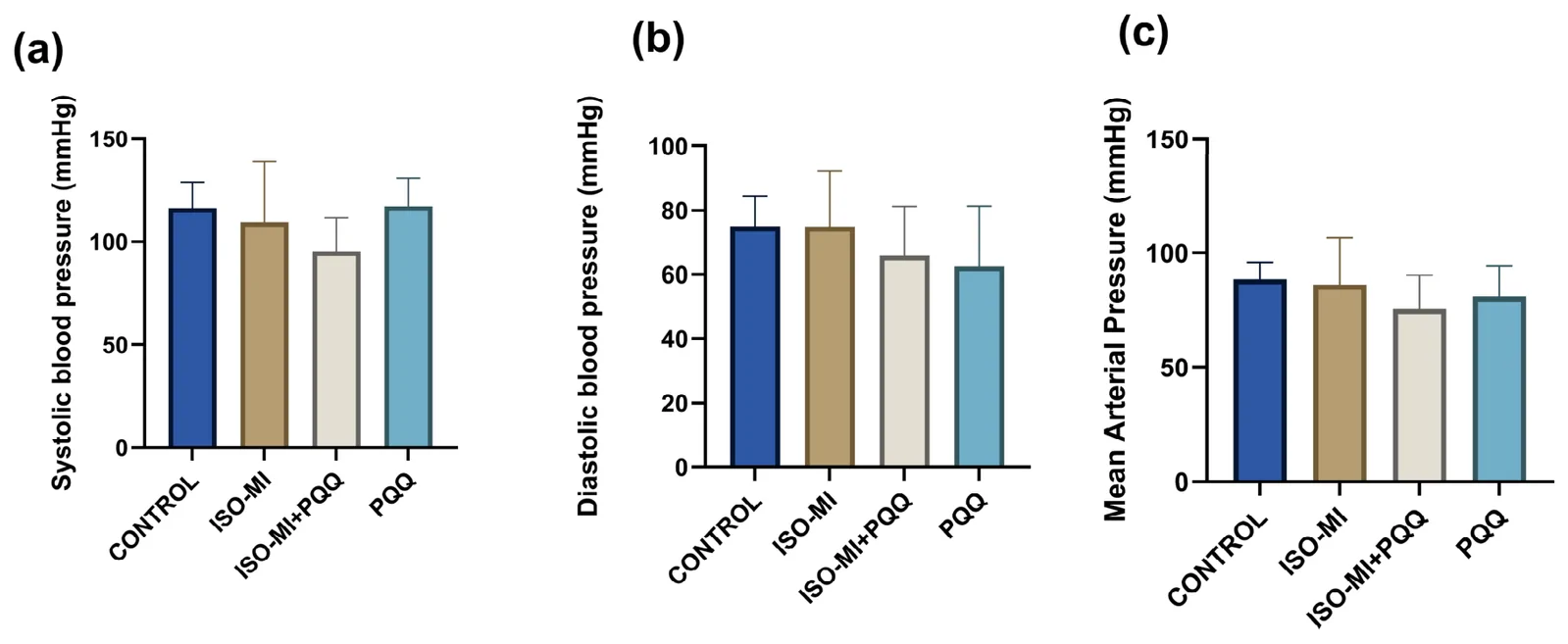
Effects of PQQ on (**a**) systolic blood pressure, (**b**) diastolic blood pressure and (**c**) mean arterial pressure in Control, IS0-MI, ISO-MI+POQ, and POQ groups. Values are represented as mean ± SEM using ANOVA followed by a post hoc Bonferroni test, respectively.

Systolic blood pressure, diastolic blood pressure and mean arterial pressure values of the Control group, ISO-MI group, ISO-MI + PQQ group and PQQ group were compared. The Blood Pressure Values are given in Table 2 and Figure 4. Overall, no significant alterations were found in hemodynamic parameters among the experimental groups.

The median (IQR) heart rate values were 157.2 (152.7–244.6) bpm in the Control group, 206.4 (162.0–235.2) bpm in the ISO-MI group, 182.6 (167.4–226.4) bpm in the ISO-MI+PQQ group, and 174.9 (143.6–235.0) bpm in the PQQ group. No statistically significant difference in heart rate was observed among the groups.

### 3.6. The Heart Tissue Histopathology Results

#### 3.6.1. Light Microscopic Findings

**Figure 5:**
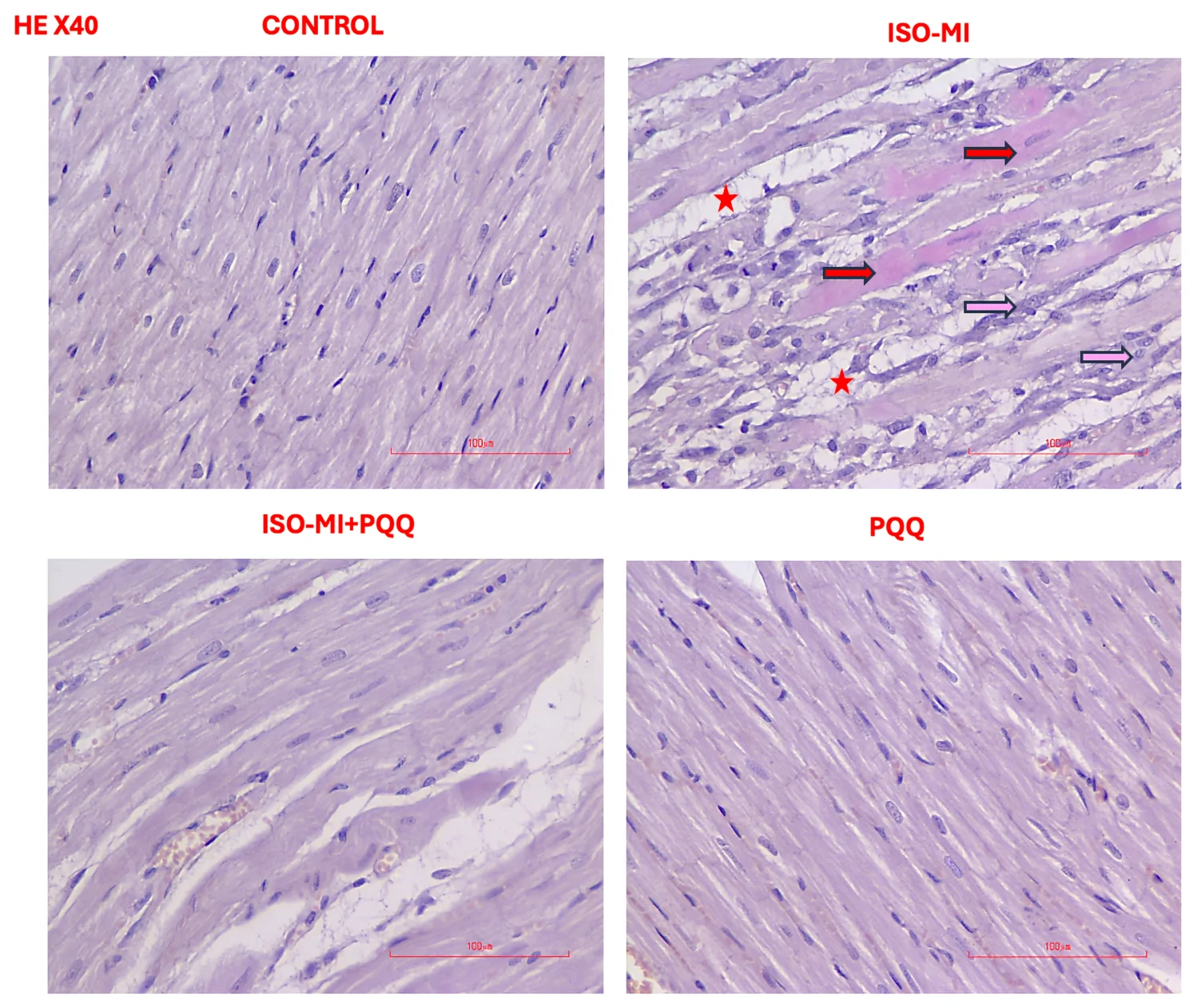
Light microscopic findings in rat cardiac tissue (Nikon Eclipse Si, Tokyo, Japan, X200 and X400). In the Control and PQQ groups, myocardial fibers with normal architecture and centrally located nuclei are observed. The HE-stained histological section of the ISO-MI group shows cardiomyocyte necrosis (red arrow), interstitial edema (red star), and inflammatory cell infiltration (pink arrow).

**Figure 6:**
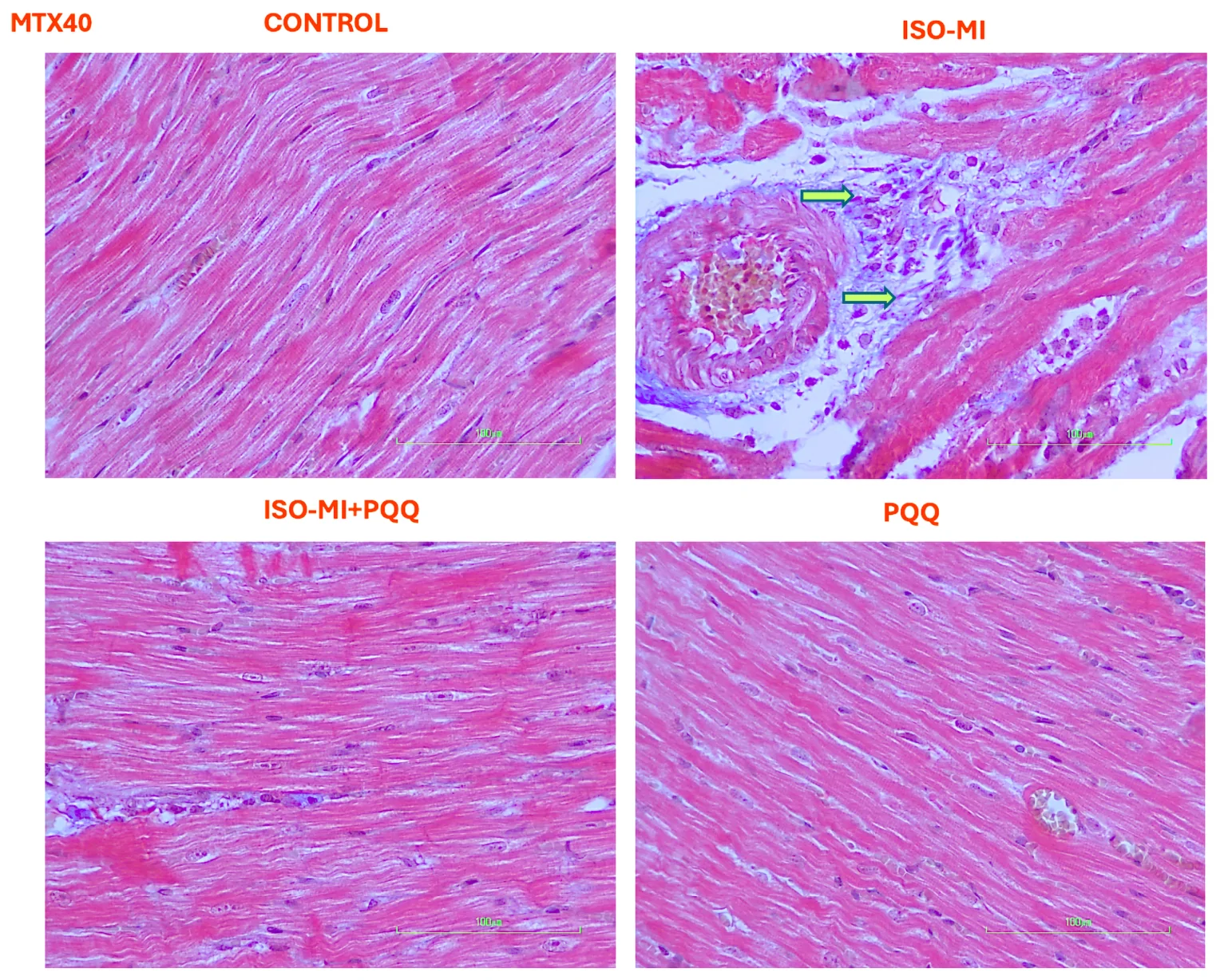
Light microscopic findings in rat cardiac tissue (Nikon Eclipse Si, Tokyo, Japan, X200 and X400). The ISO-MI group of MT stained histological section shows fibrotic scar areas (green arrow).

Light microscopic examination of cardiac tissue sections revealed normal histological architecture in the Control and PQQ groups. Cardiomyocytes were regularly arranged with preserved myocardial fibers and centrally located nuclei. The myocardial fibers maintained their normal parallel orientation and striated appearance, with no evidence of structural disruption. The overall myocardial architecture appeared intact and well organized. In contrast, cardiac sections from the ISO-MI group demonstrated marked structural alterations in myocardial tissue. Myocardial fibers exhibited loss of their normal parallel organization, with evident myofibrillar degeneration, fragmentation, and separation of muscle fibers. In addition, disruption of striations and irregular branching of cardiomyocytes were observed in affected areas. These structural abnormalities were accompanied by cardiomyocyte necrosis, interstitial edema, and infiltration of inflammatory cells within the myocardial interstitium. MT staining further demonstrated fibrotic scar formation in damaged myocardial regions. Semi-quantitative scoring revealed that these histopathological alterations were markedly increased in the ISO-MI group compared with the control and ISO-MI+PQQ groups (Table 3, Figure 5 and Figure 6).

#### 3.6.2. Immunohistochemical Findings

**Figure 7:**
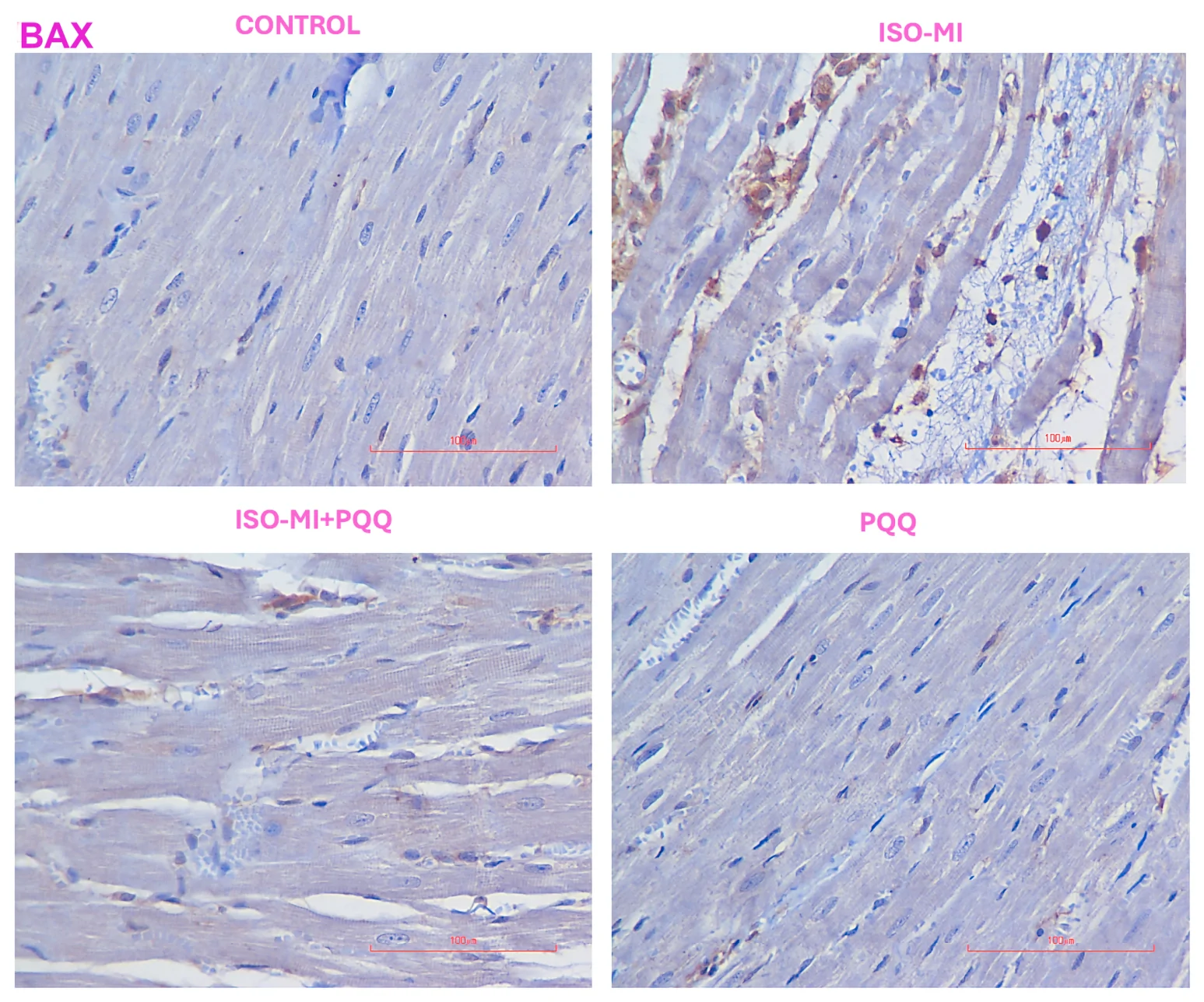
Immunohistochemical staining of BAX in rat cardiac tissue (Nikon Eclipse Si, Tokyo, Japan, X200 and X400). BAX immunoreactivity in myocardial tissue is shown in the Control, ISO-MI, ISO-MI+PQQ, and PQQ groups. Low BAX expression is observed in the Control and PQQ groups, whereas more widespread BAX-positive staining is seen in myocardial fibers in the ISO-MI group. In the ISO-MI+PQQ group, BAX immunoreactivity appears more limited compared with the ISO-MI group (*p* < 0.0001). Values are represented as mean ± SEM using ANOVA followed by a post hoc Bonferroni test, respectively.

In the Control group, BAX immunoreactivity in the myocardial tissue was observed at a low level. In the ISO-MI group, BAX-positive staining appeared more widespread and was observed as brown immunoreactivity, particularly along the myocardial muscle fibers. In addition, BAX-positive staining was also detected in some cells located within the interstitial connective tissue. In the ISO-MI+PQQ group, BAX immunoreactivity was observed in more limited areas of the myocardial tissue compared with the ISO-MI group, and the difference was statistically significant (*p* < 0.0001) (Table 4, Figure 7).

**Figure 8:**
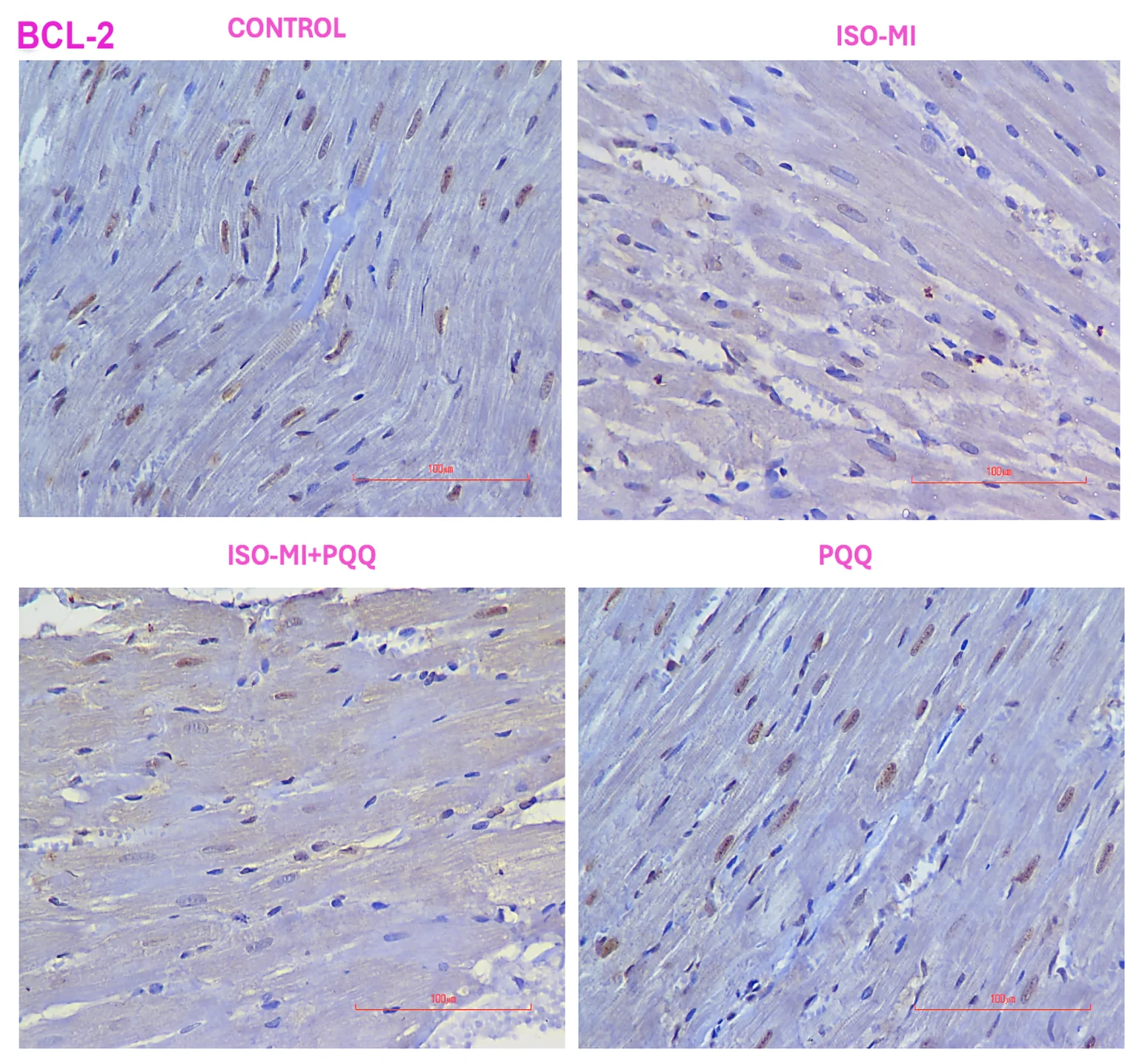
Immunohistochemical staining of BCL-2 in rat cardiac tissue (Nikon Eclipse Si, Tokyo, Japan, X200 and X400). BCL-2 immunoreactivity in myocardial tissue is shown in the Control, ISO-MI, ISO-MI+PQQ, and PQQ groups. In the Control and PQQ groups, BCL-2 expression is observed in cardiomyocytes. In the ISO-MI group, BCL-2-positive staining appears more limited in myocardial tissue. In the ISO-MI+PQQ group, BCL-2 immunoreactivity is observed in cardiomyocytes in more areas compared with the ISO-MI group; however, this difference was not statistically significant (*p* = 0.453). Values are expressed as median (min–max). Statistical analyses were performed using Kruskal–Wallis tests followed by Dunn’s post hoc analyses.

In the Control and PQQ groups, BCL-2 immunoreactivity was observed in the nuclei of cardiomyocytes within the myocardial tissue. In the ISO-MI group, BCL-2 positive staining appeared more limited within the myocardial fibers. In the ISO-MI+PQQ group, BCL-2 immunoreactivity was observed in the nuclei of cardiomyocytes and appeared in more areas of the myocardial tissue compared with the ISO-MI group. However, this change was not found to be statistically significant (*p* = 0.453) (Table 4, Figure 8).

## 4. Discussion

In the present study, the potential cardioprotective effects of PQQ against ISO-induced MI were evaluated through biochemical, histopathological, and immunohistochemical analyses. Administration of ISO successfully produced myocardial injury, as indicated by elevated cardiac injury markers, increased oxidative stress, and pronounced structural alterations in cardiac tissue. PQQ treatment markedly alleviated these pathological changes, suggesting a protective effect against myocardial damage.

Elevated Troponin T levels observed in the MI group confirmed the development of myocardial injury following ISO administration [28]. In contrast, the reduction of Troponin T levels in the ISO-MI+PQQ group indicated that PQQ may limit myocardial cell damage and reduce infarct severity. Previous experimental studies similarly demonstrated that PQQ administration decreases infarct size and suppresses lipid peroxidation during ischemic injury [19,22]. It has been reported that PQQ treatment at doses ranging from 15 to 20 mg/kg can substantially reduce infarct area in both ischemia and ischemia/reperfusion models [19].

In MI, a reduction in blood pressure is an expected hemodynamic consequence because infarction rapidly decreases cardiac output and stroke volume, impairs contractility, increases afterload, and disrupts ventriculo-arterial coupling [29]. Experimental rat MI models likewise show decreased systolic and diastolic blood pressure together with reduced left ventricular systolic pressure and ±dP/dt after infarction [30]. In the present study, although PQQ was administered with the aim of improving hemodynamic parameters, no significant differences were observed among the groups, and the lowest systolic blood pressure was detected in the ISO-MI+PQQ group. This finding does not necessarily indicate a harmful hypotensive action of PQQ, because post-MI hemodynamic responses are often nonlinear and model-dependent, and blood pressure alone may not sensitively reflect myocardial protection [31,32].

Although PQQ treatment increased SOD levels compared with the control group, this increase did not represent a significant PQQ-related improvement in the ISO-MI group. Although previous studies have reported antioxidant effects of PQQ, our findings demonstrated a significant improvement only in catalase activity, whereas SOD and MDA levels were not significantly altered [33,34,35]. These findings may differ from previous studies because the effects of PQQ appear to depend on the experimental model and cellular context. In cardiac disease models, PQQ has been reported to reduce hypertrophy, oxidative stress, and mitochondrial dysfunction and to improve cardiac remodeling or injury markers [23,35,36]. PQQ has also been reported to reduce ROS, preserve mitochondrial membrane potential, and increase antioxidant systems such as SOD2 in stressed cardiomyocytes [36,37]. These previously proposed mechanisms should therefore be regarded as hypotheses rather than mechanisms demonstrated in the present study.

Oxidative stress is a major contributor to myocardial injury, and the balance between oxidants and antioxidants is critically disrupted after myocardial infarction. Previous studies have shown that catalase, superoxide dismutase, and glutathione peroxidase activities decline markedly after myocardial infarction, thereby promoting oxidative injury and progression of tissue damage [38]. Restoration of antioxidant enzyme activities has been observed following treatment with various antioxidant compounds, including biochanin A, garlic oil, and L-2-oxothiazolidine-4-carboxylate [38,39,40]. Although transient increases in SOD expression may occur during the acute phase of myocardial injury, these elevations are generally temporary and do not necessarily correspond to sustained enzymatic activity [41]. Previous studies have suggested that the protective effects of PQQ may be related to its free radical scavenging capacity, suppression of lipid peroxidation, and preservation of mitochondrial integrity. However, these mechanisms were not directly evaluated in the present study. Furthermore, previous evidence suggests that PQQ may provide stronger mitochondrial protection against oxidative injury compared with metoprolol [22].

Histopathological examination revealed severe myocardial degeneration in the ISO-MI group, characterized by necrosis, edema, inflammatory cell infiltration, and fibrosis, all of which are typical features of ISO-induced cardiac injury [42]. PQQ treatment significantly reduced the severity of these histopathological alterations, indicating attenuation of tissue remodeling and fibrotic responses. Similar protective effects of PQQ have been reported in studies investigating cardiac hypertrophy and diabetic cardiomyopathy, where reduced fibrosis and decreased cellular injury were observed [33,43].

In the present study, BAX immunoreactivity was more prominent in the ISO-MI group and significantly reduced in the ISO-MI+PQQ group. The ISO-MI group also showed marked myocardial structural alterations, including cardiomyocyte degeneration and necrosis, interstitial edema, and inflammatory cell infiltration. These findings should be interpreted in the context of the dynamic nature of myocardial injury, as both inflammatory cell recruitment and apoptosis-related responses change during the progression and repair of myocardial damage. Previous studies have reported time-dependent changes in inflammatory cell populations and BAX expression following myocardial injury [44,45,46]. Therefore, the observed BAX positivity and inflammatory infiltration may reflect the stage of myocardial injury at the time of tissue assessment rather than static indicators of injury severity.

The novelty of this study lies in the evaluation of the effects of PQQ in an ISO-induced myocardial injury model using several complementary approaches. In the same experimental model, we assessed hemodynamic, biochemical, oxidative stress, histopathological, and apoptosis-related parameters. Thus, this study provides a multidimensional assessment of the potential cardioprotective effects of PQQ against ISO-induced myocardial injury.

The clinical translation of these findings should be considered with caution. Although PQQ showed protective effects against ISO-induced myocardial injury in rats, this experimental model does not fully reproduce the complex pathophysiology of human MI, which is most commonly caused by coronary artery occlusion and atherosclerotic plaque rupture. In addition, differences between animals and humans in PQQ pharmacokinetics, effective dose, metabolism, and treatment timing may limit the direct application of these findings to clinical practice. The present results therefore provide preclinical evidence rather than direct support for the clinical use of PQQ in patients with MI. Further studies using clinically relevant models, followed by well-designed clinical trials, are needed to determine the efficacy and safety of PQQ in humans.

## 5. Conclusions

In conclusion, the present study suggests that PQQ may exert cardioprotective effects against ISO-induced myocardial injury in rats. PQQ treatment was associated with improvements in cardiac injury biomarkers, CAT activity, histopathological findings, and reduced BAX expression. Notably, CAT activity was significantly improved, whereas MDA and SOD levels were not significantly altered, indicating that a broad antioxidant effect cannot be conclusively established based on the present findings. The reduction in BAX expression may indicate an effect on apoptosis-related processes, although the underlying mechanisms were not directly investigated in this study. Overall, these findings provide supportive evidence for the cardioprotective effects of PQQ in an ISO-induced myocardial injury model. Further studies are needed to clarify the underlying mechanisms and determine the potential clinical relevance of these findings.

## 6. Limitations

This study has several limitations. First, the findings are based on an experimental animal model that does not fully reproduce the complex pathophysiology of human myocardial infarction. Second, ventricular function, myocardial injury size, and invasive hemodynamic parameters were not assessed. Although biochemical, histopathological, immunohistochemical, and non-invasive hemodynamic parameters were evaluated, these assessments do not directly measure ventricular function or quantitatively determine the extent of myocardial injury. Third, the relatively small sample size, with eight animals initially included in each experimental group, may have limited the statistical power to detect smaller effects. Although the sample size was based on a previously published study using a similar experimental model, no formal a priori sample size calculation or statistical power analysis was performed. Therefore, both statistically significant and non-significant findings should be interpreted with caution. In particular, the relatively small sample size may have limited the ability of the Shapiro–Wilk test to detect departures from normality, and the results of the normality assessment should therefore be interpreted cautiously.

Fourth, only a limited set of oxidative stress and apoptosis-related markers was evaluated, and the molecular mechanisms underlying the potential cardioprotective effects of PQQ were not directly investigated. Therefore, the observed changes in CAT activity and BAX expression should not be interpreted as evidence of a specific underlying molecular mechanism. Fifth, histopathological assessment was performed by a single investigator who was blinded to the experimental groups; however, intra-observer reproducibility of the semi-quantitative histopathological scoring was not formally assessed. This should be considered a methodological limitation. Finally, long-term outcomes and dose–response relationships were not investigated. Therefore, the physiological and clinical relevance of the present findings should be interpreted with caution. Future studies incorporating echocardiographic assessment, quantitative evaluation of myocardial injury, invasive hemodynamic measurements, additional molecular analyses, and different doses and treatment durations are needed to further clarify the cardioprotective effects of PQQ.

## Figures and Tables

**Table 1:** Biochemical parameters assessed in this study.

	Control	ISO-MI	ISO-MI+PQQ	PQQ	*p* Values
Troponin T	16.1 ± 0.8 ^a^	24.6 ± 2.7 ^b^	17.7 ± 1.6 ^c^	18.9 ± 1.0	a–b, *p* = 0.01; b–c, *p* = 0.04
BNP	359.8 ± 110.2	481.9 ± 117.8	492.7 ± 145.3	481.9 ± 109.0	*p* > 0.05
MDA	12.9 ± 0.8	13.5 ± 0.8	11.89 ± 0.5	11.8 ± 0.9	*p* > 0.05
SOD	159.9 ± 2.7 ^a^	178.7 ± 2.4 ^b^	181.0 ± 1.1 ^c^	173.9 ± 2.0 ^d^	a–b, *p* < 0.0001; a–c *p* < 0.0001; a–d; *p* = 0.0008
CAT	164.6 ± 8.9 ^a^	97.6 ± 17.3 ^b^	156.3 ± 18.9 ^c^	176.2 ± 10.0 ^d^	a–b; *p* = 0.01, b–c; *p* = 0.04 b–d; *p* = 0.002

Values are presented as mean ± SEM. One-way ANOVA with Bonferroni’s post hoc test was used. Different superscript letters indicate significant differences (*p* < 0.05).

**Table 2:** Blood Pressure Parameters in Experimental Groups (Mean ± SEM).

	Control	ISO-MI	ISO-MI+PQQ	PQQ
Systolic Blood Pressure Values	116.5 ± 4.6	109.3 ± 10.5	95.3 ± 5.8	117.3 ± 4.7
Diastolic Blood Pressure Values	74.9 ± 3.6	74.8 ± 6.2	65.8 ± 5.5	62.5 ± 6.7
Mean Arterial Pressure Values	88.7 ± 2.7	86.3 ± 7.3	75.6 ± 5.2	80.8 ± 4.8

**Table 3:** Cardiac histopathological scores in experimental groups.

	Control	ISO-MI	ISO-MI+PQQ	PQQ	*p* Values
Cardiomyocyte necrosis scoring	0.0 (0.0–1.0) ^a^	3.0 (1.0–3.0) ^b^	1.0 (0.0–2.0) ^c^	0.0 (0.0–1.0) ^d^	a–b, *p* < 0.0001; b–c, *p* < 0.0001
Interstitial edema	0.0 (0.0–1.0) ^a^	3.0 (1.0–3.0) ^b^	1.0 (0.0–3.0) ^c^	0.0 (0.0–1.0) ^d^	a–b, *p* < 0.001; b–c, *p* < 0.001
Inflammatory cell infiltration	0.0 (0.0–1.0) ^a^	3.0 (2.0–3.0) ^b^	1.0 (0.0–2.0) ^c^	0.0 (0.0–2.0) ^d^	a–b, *p* < 0.001; b–c, *p* < 0.001
Fibrotic scar areas	0.0 (0.0–1.0) ^a^	3.0 (2.0–3.0) ^b^	0.0 (0.0–2.0) ^c^	0.0 (0.0–1.0) ^d^	a–b, *p* < 0.001; b–c, *p* < 0.001

Values are expressed as median (min–max). Statistical analyses were performed using Kruskal–Wallis tests followed by Dunn’s post hoc analyses. Different superscript letters indicate significant differences between groups (*p* < 0.05).

**Table 4:** Immunoreactivity intensity of apoptosis markers in experimental groups.

	Control	ISO-MI	ISO-MI+PQQ	PQQ	*p* Values
**BAX**	78.9 (71.0–89.0) ^a^	83.5 (75.5–97.9) ^b^	76.6 (67.585.5) ^c^	81.0 (78.2–96.4) ^d^	a–b, *p* = 0.00086 b–c, *p* < 0.0001 c–d, *p* = 0.0005
**BCL-2**	81.7 (73.3–97.7)	84.1 (73.3–91.3)	80.2 (67.5–93.6)	86.5 (74.2–101)	*p* > 0.05

Values are presented as median (min–max). Kruskal–Wallis test followed by Dunn’s post hoc test was used. Different superscript letters indicate significant differences between groups (*p* < 0.05).

## Data Availability

All relevant data generated or analyzed during this study are included in this published article. Further details are available from the corresponding author on request.

## Abbreviations

ISO	Isoproterenol
PQQ	Pyrroloquinoline quinone
MI	Myocardial infarction
CVDs	Cardiovascular diseases
IHD	Ischemic heart disease
ROS	Reactive oxygen species
ELISA	Enzyme-linked immunosorbent assay
BNP	B-type Natriuretic Peptide
MDA	Malondialdehyde
SOD	Superoxide Dismutase
CAT	Catalase
DAB	Diaminobenzidine
H&E	Hematoxylin and eosin
MT	Masson’s Trichrome
